# A simple solid media assay for detection of synergy between bacteriophages and antibiotics

**DOI:** 10.1128/spectrum.03221-23

**Published:** 2024-03-25

**Authors:** Ethan Khong, Joseph J. Oh, Julian M. Jimenez, Roland Liu, Sage Dunham, Alisha Monsibais, Alison Rhoads, Pooja Ghatbale, Andrew Garcia, Ana Georgina Cobián Güemes, Alisha N. Blanc, Megan Chiu, Peiting Kuo, Marissa Proost, Ahnika Kline, Saima Aslam, Robert T. Schooley, Katrine Whiteson, Stephanie I. Fraley, David T. Pride

**Affiliations:** 1 Department of Pathology, University of California San Diego, La Jolla, California, USA; 2 Department of Bioengineering, University of California San Diego, La Jolla, California, USA; 3 Department of Molecular Biology and Biochemistry, University of California Irvine, Irvine, California, USA; 4 Department of Medicine, University of California San Diego, La Jolla, California, USA; University of Exeter, Exeter, United Kingdom

**Keywords:** synergy, cooperativity, antibiotics, bacteriophages, solid media

## Abstract

The emergence of antibiotic-resistant bacteria (ARB) has necessitated the development of alternative therapies to deal with this global threat. Bacteriophages (viruses that target bacteria) that kill ARB are one such alternative. Although phages have been used clinically for decades with inconsistent results, a number of recent advances in phage selection, propagation, and purification have enabled a reevaluation of their utility in contemporary clinical medicine. In most phage therapy cases, phages are administered in combination with antibiotics to ensure that patients receive the standard-of-care treatment. Some phages may work cooperatively with antibiotics to eradicate ARB, as often determined using non-standardized broth assays. We sought to develop a solid media-based assay to assess cooperativity between antibiotics and phages to offer a standardized platform for such testing. We modeled the interactions that occur between antibiotics and phages on solid medium to measure additive, antagonistic, and synergistic interactions. We then tested the method using different bacterial isolates and identified a number of isolates where synergistic interactions were identified. These interactions were not dependent on the specific organism, phage family, or antibiotic used. *A priori* susceptibility to the antibiotic or the specific phage were not requirements to observe synergistic interactions. Our data also confirm the potential for the restoration of vancomycin to treat vancomycin-resistant Enterococcus (VRE) when used in combination with phages. Solid media assays for the detection of cooperative interactions between antibiotics and phages can be an accessible technique adopted by clinical laboratories to evaluate antibiotic and phage choices in phage therapy.

**IMPORTANCE:**

Bacteriophages have become an important alternative treatment for individuals with life-threatening antibiotic-resistant bacteria (ARB) infections. Because antibiotics represent the standard-of-care for treatment of ARB, antibiotics and phages often are delivered together without evidence that they work cooperatively. Testing for cooperativity can be difficult due to the equipment necessary and a lack of standardized means for performing the testing in liquid medium. We developed an assay using solid medium to identify interactions between antibiotics and phages for gram-positive and gram-negative bacteria. We modeled the interactions between antibiotics and phages on solid medium, and then tested multiple replicates of vancomycin-resistant Enterococcus (VRE) and Stenotrophomonas in the assay. For each organism, we identified synergy between different phage and antibiotic combinations. The development of this solid media assay for assessing synergy between phages and antibiotics will better inform the use of these combinations in the treatment of ARB infections.

## INTRODUCTION

The rise in antibiotic-resistant bacteria (ARB) has become a global public health issue that threatens the lives of millions of people across the world every year (1). Among ARB, the ESKAPE pathogens (*Enterococcus faecium*, *Staphylococcus aureus*, *Klebsiella pneumoniae*, *Acinetobacter baumannii*, *Pseudomonas aeruginosa*, and *Enterobacter* spp.) are often multidrug resistant and are the leading cause of nosocomial infections. One potential solution to the growing threat of ARB is the use of bacteriophages (viruses that attack and kill bacteria) as alternative treatments to antibiotics. Thus, phage therapy utilizing these bacteria-targeting virus so far has largely been reserved for treatment of bacterial infections that are highly resistant to antibiotics (2) but could potentially have broader applications. There have been successful outcomes in a number of recent phage therapy cases (3).

Antibiotics are the current standard-of-care for the treatment of ARB infections. Since phages have not yet received regulatory approval, they are usually delivered in conjunction with antibiotics to ensure that the standard-of-care is met. When used in combination with antibiotics, it is difficult to determine the contributions of each to the eradication of the infection. In general, the field lacks randomized clinical trials to determine whether these combination therapies are effective (4, 5). One of the first steps toward determining whether these combination therapies can be effective is to investigate whether there are cooperative or even antagonistic interactions between antibiotics and phages in *in vitro* systems. The lack of a standardized, accessible assay for determining cooperativity limits the field significantly.

The current methodology for determining whether there may be cooperative effects between antibiotics and phages is performed primarily in broth medium, where the target ARB is cultivated in the presence of antibiotic and phage. There are different methodologies to perform these broth assays (6–8), but no single procedure is universally accepted. Additionally, these assays are highly complex for clinical laboratory personnel, who need extensive training, and the assays require the acquisition of expensive equipment such as microplate readers (9–11). Because of the extensive changes that would need to occur to bring such broth-based assays into use in clinical microbiology facilities across the globe, we sought to examine whether there might be alternative means for examining cooperativity between phages and antibiotics without the need for the purchase of complex or expensive equipment. Although there have now been several studies to examine the cooperative phenomena between antibiotics and phages in broth (12), relatively little has been done to identify whether such relationships can be demonstrated on solid medium.

To address a growing need to understand the effects of the combination of antibiotics and phages against ARB, we sought to develop a cooperativity assay on solid medium. Such an assay can be performed without expensive equipment and has the potential to provide results that can be interpreted in a simplified fashion by comparing the observed bacteria clearance patterns with the predicted patterns of cooperativity. Our goals were to (i) develop an assay that can be easily performed in most clinical laboratories, (ii) determine whether cooperative interactions between antibiotics and phages occur on solid medium for gram-positive and gram-negative bacteria, (iii) decipher whether susceptibility to certain antibiotics and/or phages is necessary to demonstrate cooperativity, and (iv) provide a template for straightforward interpretation of results without the need for mathematical modeling of antibiotics and phages diffusion on each assay.

## MATERIALS AND METHODS

### Transient diffusion in a semi-infinite medium approximation

A custom MATLAB (MathWorks, Inc) script was developed to model the diffusion of antimicrobial agents (antibiotic drug or phage) through an agarose medium. To model the perpendicular strips placed on an agarose plate, the concentration profiles of two agents diffusing perpendicular to each other were calculated. The semi-infinite approximation for diffusive mass transfer was used as previously described (13, 14) to predict the concentration of two agents: antibiotic (α) and phage (β), C_α_(x,t) and C_β_(y,t), as a function of distance and time (equation 1A, 1B) (Tables S1 and S2). The error function (equation 2A, 2B) and non-dimensionalized distance (equation 3A, 3B) were utilized to solve for the concentrations at each iterative distance and time interval. The following simplifying assumptions were made: one-dimensional diffusion, dilute solution upon contact with agarose, transient diffusion. The concentrations C_α,source_ and C_β,source_ μg/mL were defined as an infinitely abundant sources C_α_(0,t) for x = 0 cm and C_β_(0,t) for y = 0 cm, respectively. The initial concentration C0 of all other points was defined as 0 µg/mL for C_α_(x,0) and C_β_(y,0). By assuming that the agents diffuse a minute distance during the finite time of exposure relative to the size of the plate, we apply the semi-infinite medium approximation and set a boundary condition such that C_α_(∞,0) and C_β_(∞,0) = C_0_.

### Predicting drug interactions for equal concentration and equal diffusion coefficients

Using the semi-infinite medium approximation, contour plots of the concentration profile at different times were plotted on a 3 cm × 3 cm grid. Initially, agents α and β were modeled using equal source concentrations C_α,source_ = C_β,source_ = 1.0 μg/mL and diffusion coefficient D_α_ = D_β_ = 1 × 10^−6^ cm^2^/s (15, 16) on the order of magnitude for an antibiotic drug diffusing through agarose. Different potential interactions between agents α and β were considered. No interaction between agents was modeled using the highest-single agent (HSA) model (17) (equation 4) (Table S3). This assumes that each agent acts independently, and the antibiotic effect of the combined agents is dictated by the higher concentration. Additive interactions were modeled following the assumption from the Loewe Additive Interaction model (17), i.e., concentrations of each individual agent can be added together as if they were the same agent (equation 5). Synergistic interactions are defined as interactions that result in a higher effect than an additive interaction (17). Synergistic interactions were modeled such that the effective concentration is the additive concentration plus the product of the concentrations, which is modulated by a coefficient k (equation 6). Antagonistic interactions are defined as interactions that result in a lower effect than the additive interaction (17). Antagonistic interactions were modeled using the assumption that each antibiotic agent is mutually antagonistic, with the overall effective concentration modulated by a coefficient q (equation 7). Minimum bactericidal concentration (MBC) curves were plotted over the concentration contours to visualize the resultant live bacterial lawn profiles.

### Predicting agent interactions for specific antibiotic drugs and phage combinations

Predictions for specific antibiotic (α) and phage (β) combinations were performed using the models above. However, parameters representative of the experimental conditions were used: C_α,source_ = 1.5 µg/mL and D_α_ = 1 × 10^−6^ cm^2^/s (15, 16) (for vancomycin), and C_β,source_ = 1.2 × 10^−2^ μg/mL and D_β_ = 5 × 10^−8^ cm^2^/s (18) (for phage Ben). Phage concentrations were converted from plaque forming units (PFU)/mL to μg/mL by assuming that a PFU contains an average of 1 phage (19), multiplying by the estimated molecular weight of an individual T4 phage, e.g., myovirus morphology (20, 21), and converting to mass using Avogadro’s number (Table S4).

### Bacteria, phages, and culture conditions

Bacterial strains, including isolates of VRE (vancomycin-resistant Enterococcus), VSE (vancomycin-susceptible Enterococcus), and STM (*Stenotrophomonas maltophilia*), were collected from the UCSD Center for Advanced Laboratory Medicine, under IRB#160524. All specimens collected were de-identified in such a manner that they could not be re-identified prior to their use in this study. Information, including antibiotic susceptibilities and speciation for each microbe, was recorded (Tables S5 and S6). All isolates were identified to the species level using MALDI-TOF (Brucker, Billerica, MA, USA), and antimicrobial susceptibilities using microbroth dilution on the BD Phoenix using panels PMIC-107 for gram positives and NMIC-307 for gram negatives. All the strains of bacteria and phages were cultivated in liquid brain heart infusion (BHI) medium at 37°C with shaking at 250 rpm. BHI plates were made with an equal volume of 20 mL of BHI broth infused with 1.5% agar. All phages used in this study were previously isolated and purified from environmental sources using multiple enrichment protocols as described above (22).

### Preparation of antibiotic and phage strips

Grade 1 Whatman filter paper (VWR, Visalia, CA, USA; CAT no. 1001-150) was used to make a 5 mm × 28 mm paper strip cutout using Cricut Explore Air 2. The strips were autoclaved and then soaked in prepared antibiotic stock solutions matching the antibiotic concentrations of the standard antibiotic disks. For example, for vancomycin, the strips were soaked in 1.4 mg/µL concentration of a vancomycin stock solution. Standard antibiotic stock concentrations used in this study are listed (Table S7). Similarly, phage strips were prepared using high titer (10^8^ PFU/mL) phage stock. All the antibiotic and phage strips were soaked in their corresponding solutions for 12 hours at 4°C. The strips were dried in a biosafety cabinet for 1 hour without light exposure. The dried strips were used within 1 hour of drying or were stored at 4°C for up to 12 hours before use.

### Plating, stamping, and interpretation

For each isolate, an overnight culture was diluted to 0.2 OD_600_ and was incubated for 15 min with shaking at 37°C. Then, 100 uL of culture was combined with 3 mL of warmed 0.3% top agar (BHI broth with 0.3% agar) and poured over 1.5% BHI agar plate evenly to make a bacterial lawn. The plates with bacterial lawn were dried for 2 hours at room temperature before performing a stamping procedure with both phage and antibiotic strips aligned at a 90-degree angle on a predesigned L-shape stamp (Fig. S1). The dried plates with the bacterial lawns were then inverted with the cover off and gently lowered on the L-shape stamps until the top agar was pressed minimally against the aligned strips. Then, the stamped plates facing upward were incubated at 37°C with no shaking for 18–20 hours. Control plates were prepared in a similar manner using the same stocks of antibiotic- or phage-impregnated strips. The control plates also contained blank autoclaved strips along with antibiotic disks (at the concentrations specified in Table S7) and a 4-uL spot of liquid phage stock placed directly onto the agar plate. After 18–20 hours of incubation, the plates were then imaged and analyzed.

## RESULTS

### Development of a solid media phage/antibiotic cooperativity assay

**Fig 1:**
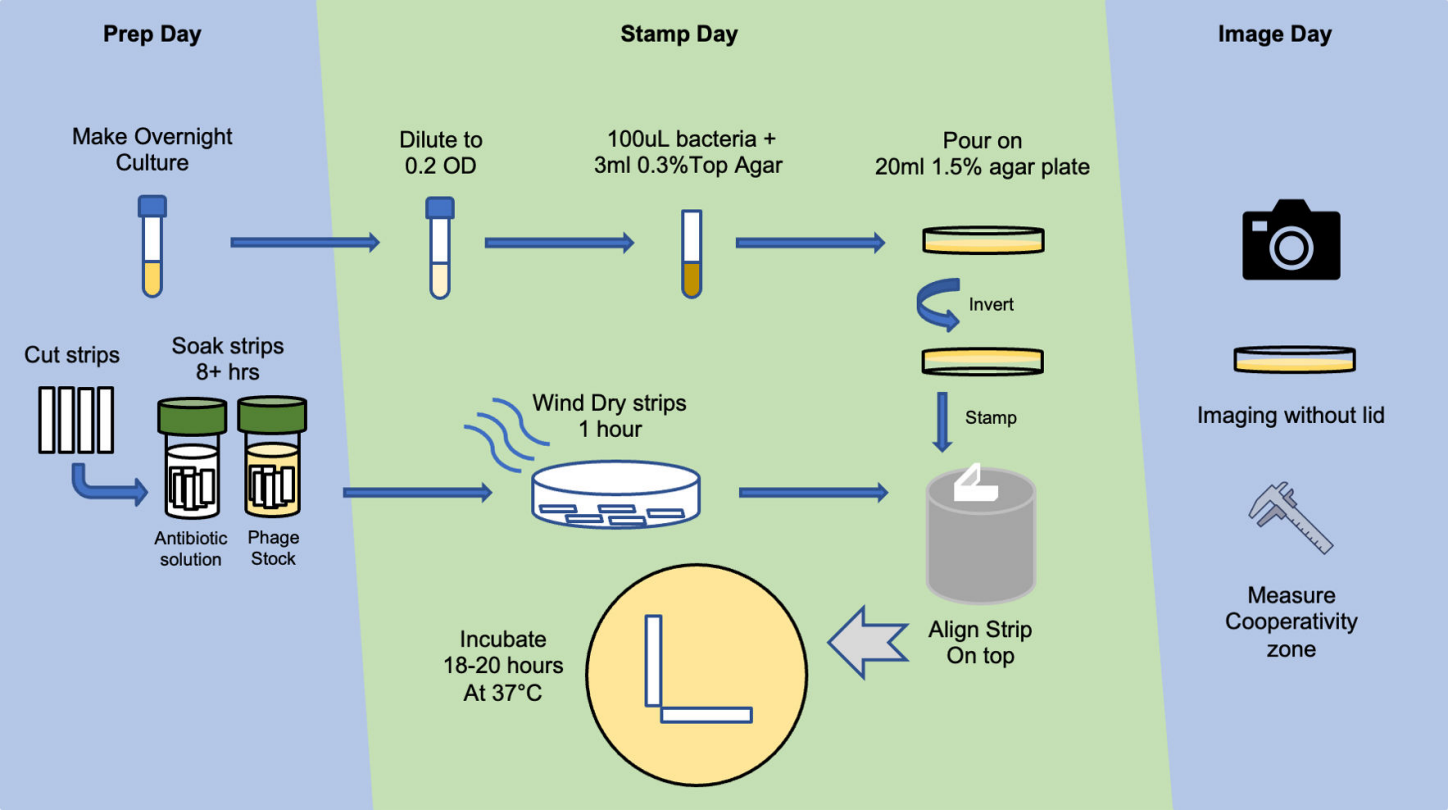
Workflow for phage–antibiotic cooperativity assays.

Our solid medium cooperativity assay design is based on the principle of impregnating separate filter paper strips with antibiotics and phages, placing the strips at a right angle on a lawn of bacteria, and then measuring growth inhibition along each strip (Fig. 1; Fig. S2). If there is cooperativity between the phage and the antibiotic, a zone of growth inhibition will form at the right angle created by the antibiotic- and phage-impregnated strips (Fig. S3).

### Measuring additivity, cooperativity, and antagonism

We developed a custom model to predict the effective concentrations of antibiotics and phages as they diffuse away from their source strips through the agar medium and interact with the bacterial lawn. We did so using the semi-infinite medium approximation for unsteady-state mass transfer (13, 14), which predicts the concentration profiles of the antibiotic and phage as a function of distance from the strips and time (Table S1). We assumed that the depth of the medium was negligible compared to the width and only modeled diffusion in the top plane of view of the plate, setting boundary conditions and parameter values for the semi-infinite medium approximation based on a combination of measured, estimated, and literature values (Table S2).

**Fig 2:**
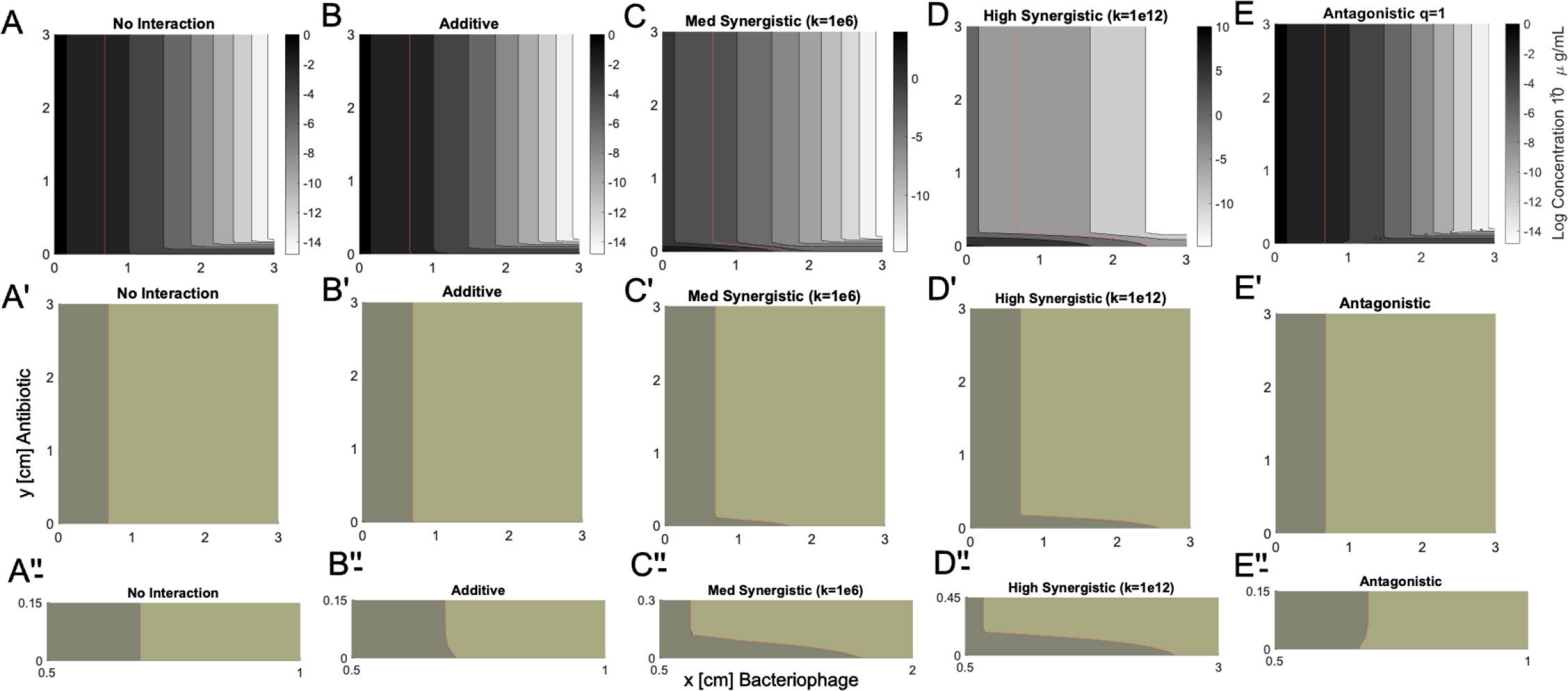
Model with parameters predictive of experimental results. Prediction of antibiotic (e.g., vancomycin) and phage (e.g., Ben) profiles based on different potential interactions. Concentration contour plots for representative antibiotic (C_α_ = 1.5 µg/mL and D_α_ = 1 x 10^−6^ cm^2^/s) and phage (C_β_ = 1.2 × 10^−2^ µg/mL and D_β_ = 5 x 10^−8^ cm^2^/s). (**A**) No interaction, (**B**) additive, (**C**) synergistic “medium” k = 1e6; (**D**) synergistic “high” k = 1e12; (**E**) antagonistic q = 1. Assuming MBC = 0.1 µg/mL (red). Panels A’–E’ and A”–E” represent magnifications of portions of the panels shown in panels A–E, respectively.

**Fig 3:**
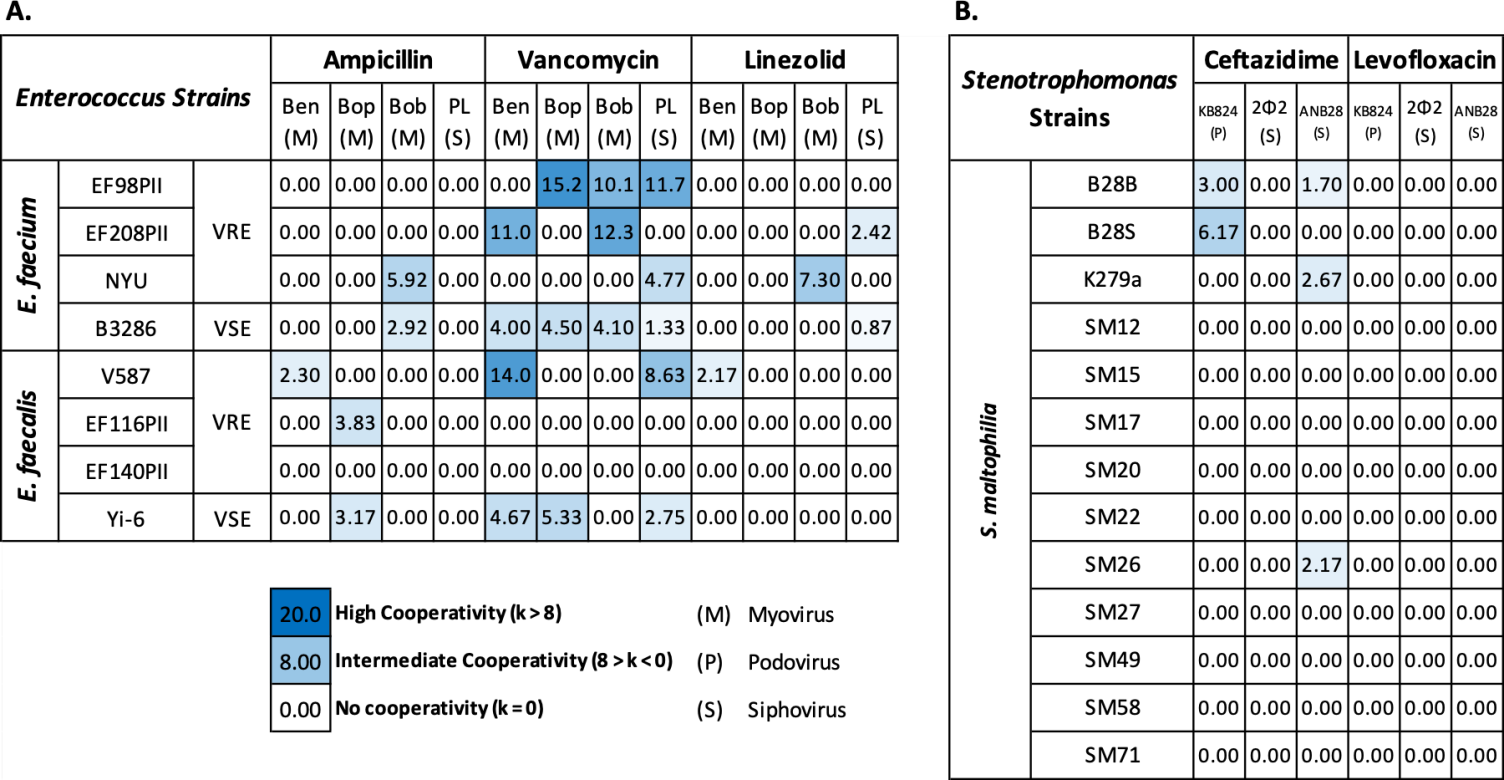
Summary heat map of all used combinations of bacteria, phage, and antibiotics evaluated for cooperativity. K-values were calculated for each experiment based on data of three biological replicates. (**A**) *E. faecium* and *E. faecalis* strains using phages Ben, Bop, Bob, or PL with antibiotics ampicillin, vancomycin, or linezolid. (**B**) *Stenotrophomonas maltophilia* strains using phages KB824, 2ϕ2, or ANB28 with antibiotics ceftazidime or levofloxacin.

We developed this model based upon the concept that we could observe killing of the bacterial lawn in areas distal to the antibiotic- or phage-impregnated strips, which would reflect an effective MBC (Fig. S4). The interface between live and dead bacteria would create a profile that aligns with the MBC that is achieved by the combinatory antibiotic and phage effect. We then could develop a computational model to predict the concentrations of the antibiotic and phages as they diffuse across the agar using contour plots that represent different experimental results (Fig. 2). Model parameters representative of the experimental conditions (Table S2) were used to predict the bacterial lawn profile under different antibiotic and phage interactions (Table S3) after 20 hours of incubation assuming an MBC of 0.1 µg/mL. k and q are tunable variables that represent different extents of synergistic or antagonistic interactions (Table S2). We performed such simulations using a gram-positive model organism, *Enterococcus* spp., and several different Enterococcus phages (Table S4). Initial concentration and coefficients of diffusion representative of vancomycin (Cα = 1.5 µg/mL and Dα = 1 × 10^–6^ cm^2^/s) and Enterococcus phage Ben (Cβ = 1.2 × 10^–2^ µg/mL and Dβ = 5 × 10^–8^ cm^2^/s) were used. We modeled no interaction (Fig. 2, panel A) and additive interactions between antibiotic and phage (Fig. 2, panel B). Our models displayed distinct convex curvatures that were indicative of strongly synergistic interactions (Fig. 2, panels C and D). For example, the model has different convex curvatures based on the extent of synergy displayed, with at least 1e6 greater killing (Fig. 2, panel C) or 1e12 greater killing (Fig. 2, panel D). Synergy mentioned here refers to multiplicative cooperativity where the antibiotic and phage combination kills more than each individual antimicrobial would be predicted to kill when combined together. All observed combinations of antibiotic and phage cooperativity were simulated to generate k-values and were visualized on a summary heat map (Fig. 3). We also could model antagonistic interactions between antibiotics and phage, which demonstrated concave curvatures (Fig. 2, panel E).

### Evaluation of cooperativity in VRE

**Fig 4:**
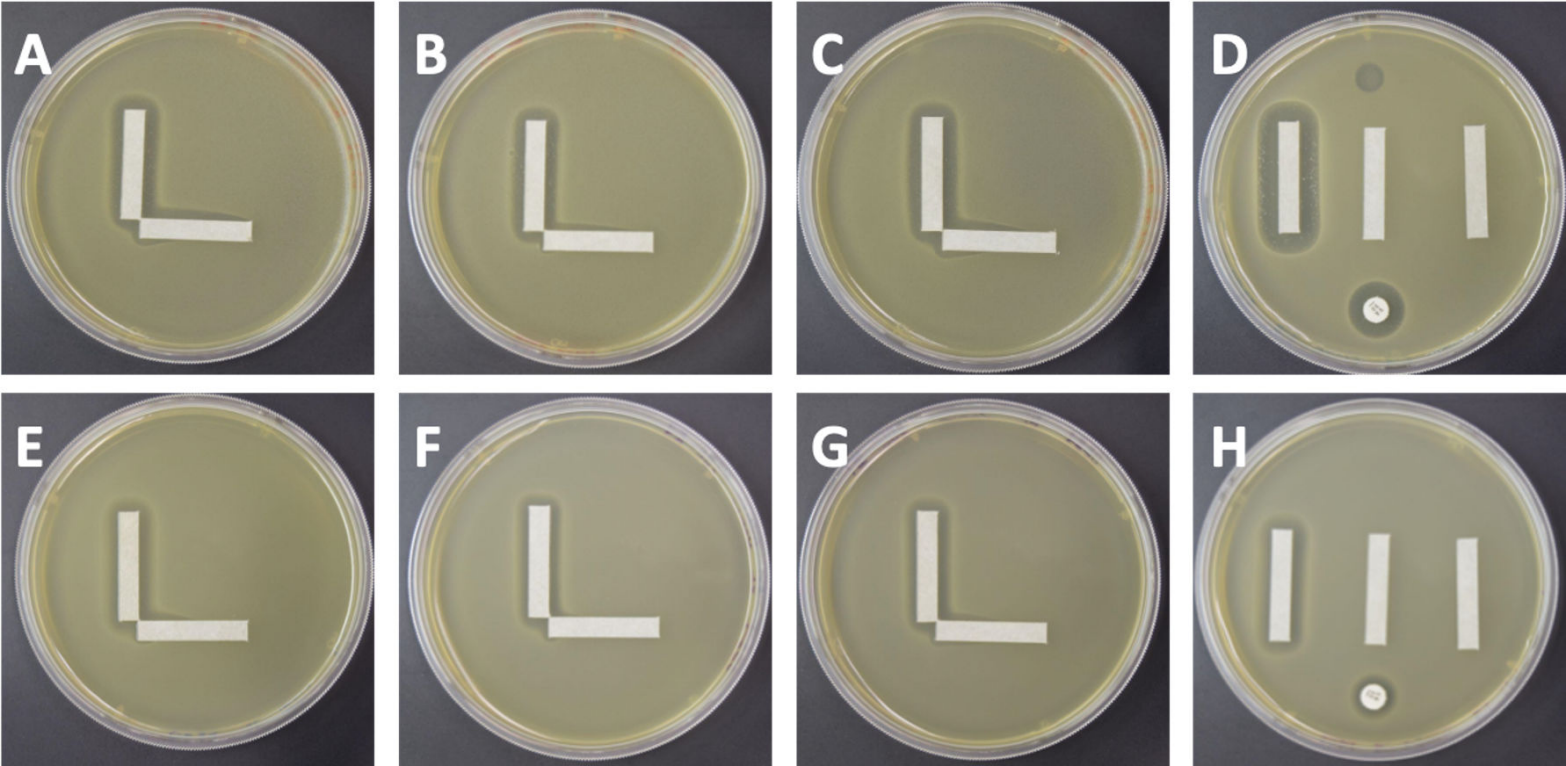
Solid media cooperativity assays for VRE. Each specimen was tested with vancomycin (vertical strip) and a phage (horizontal strip). *E. faecium* EF98PII (VRE) with phage Bop is demonstrated in panels A–D, where A–C represent three separate replicates of the cooperativity assay, and panel D represents the control plate with a vertical vancomycin strip (left), blank strip (middle), phage strip (right), antibiotic disk (bottom), and phage spot (top). *E. faecalis* V587 (VRE) with phage Bop is demonstrated in panels E–H, where panels E–G represent separate replicates and panel H represents the control plate.

We next set up this solid media cooperativity assay to determine whether we could observe patterns similar to those predicted in the model (Fig. 2). We expected to observe additional killing at the right angle where concentrations of the phage and antibiotic may be below the MBC of each individual phage or antibiotic, but together show cooperativity (Fig. S3). A separate stamping device/procedure was developed to allow for the placement of the antibiotic and phage strips at perfect right angles on the medium (Fig. S1). Each experiment was performed in triplicate to verify the accuracy and reproducibility of the results (Fig. 4).

We performed solid media cooperativity experiments for isolates of both *E. faecium* and *Enterococcus faecalis* (Fig. 3; Table S5). We chose a set of phages that were selectively active against a number of Enterococcus isolates (Table S8; genomes and further information about phage sources are available in Armstrong et al. [23]). These isolates (for both species) may become resistant to vancomycin through expression of genes for enzymes that alter cell wall amino acid composition, often contained on a plasmid (24). *E. faecium* strains EF98PII, EF208PII, and NYU, and *E. faecalis* strains V587, EF116PII, and EF140PII were determined to be vancomycin resistant based on antimicrobial susceptibility testing (Table S5). We first used the cooperativity assay to examine a highly antibiotic-resistant VRE isolate of *E. faecium* (EF98PII). We set the assay up with vancomycin as the antibiotic and Bop (myovirus) as the phage (Fig. 4). Although EF98PII is susceptible to Bop, it does not demonstrate complete lysis (Fig. 4, panel D). There is significant evidence in each of the replicates of a cooperativity zone between vancomycin and the phage (Fig. 4, panels A–C). We also identified similar interactions when *E. faecalis* was used rather than *E. faecium*, indicating that the cooperativity in VRE is not a species-specific phenomenon (Fig. 4, panels E–H).

**Fig 5:**
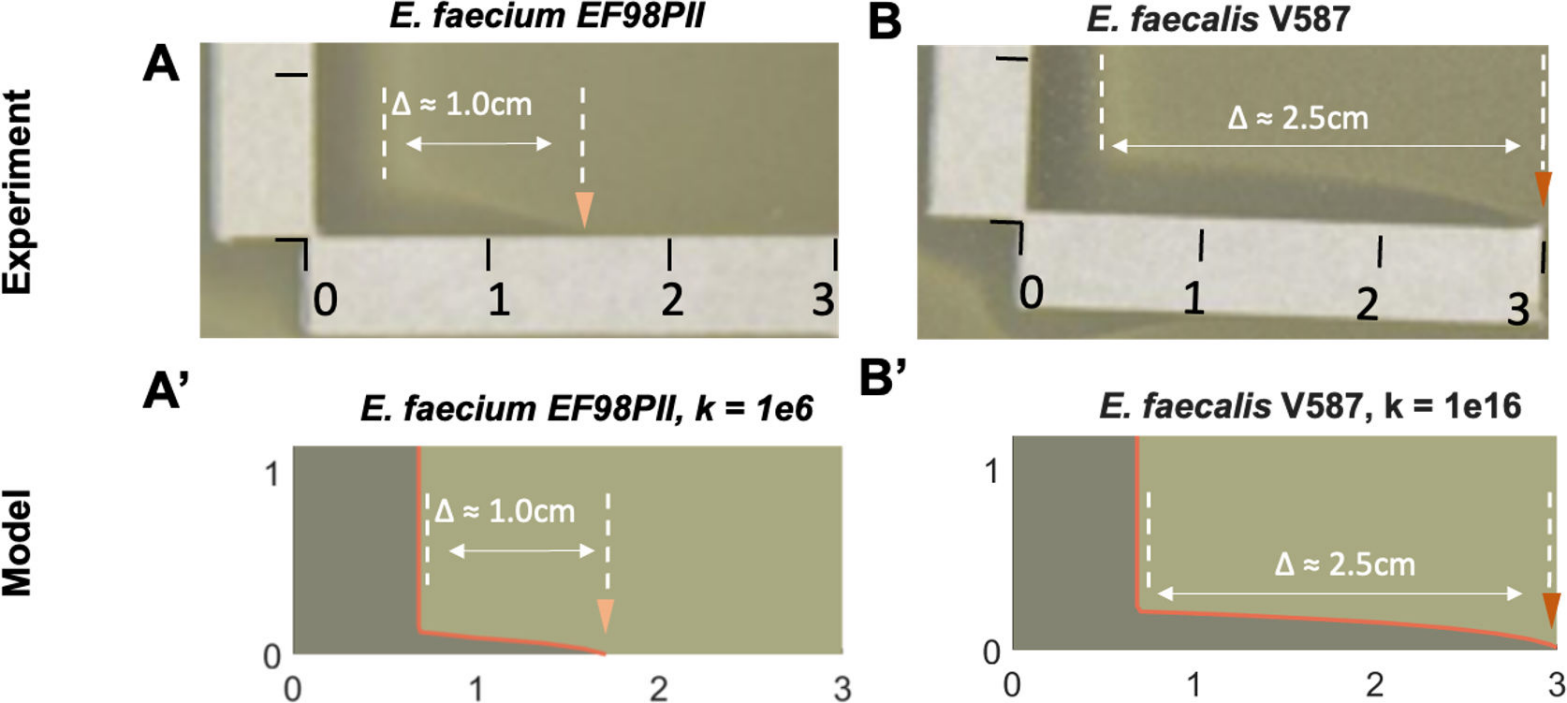
Comparison of experimental results and model predictions. (**A**) *E. faecium* EF98PII (VRE) treated with vancomycin (vertical strip) and phage Bop (horizontal strip). This resulted in a synergistic profile that extended 1.0 cm from the leading edge of the vertical zone of inhibition. (**B**) *E. faecalis* V587 (VRE) treated with vancomycin (vertical strip) and phage Bop (horizontal strip). This resulted in a synergistic profile that extended 2.5 cm from the leading edge of the vertical zone of inhibition. Model predictions for *E. faecium* EF98PII (**A’**) and *E. faecalis* V587 (**B’**) showed similar synergistic profile extensions and dimensions when the synergy coefficient was adjusted from medium synergy (k = 1e6) to high synergy (k = 1e16).

We further examined the synergistic interactions observed for vancomycin and phage Bop for the *E. faecium* and *E. faecalis* VRE isolates (Fig. 4). By measuring the extension of the zone of inhibition for *E. faecium* EF98PII, we were able to estimate the synergy coefficient (“k”) for vancomycin and phage Bop. Our results indicate that k = 1e6 (Fig. 5, panel A), which matched our model for medium-level synergistic interactions between the phage and antibiotic. For *E. faecalis* V587, the coefficient was 1e16 for vancomycin and Bop (Fig. 5, panel B), indicating that high-level synergy was observed. These data confirm that synergistic interactions occur between the antibiotic vancomycin and phage Bop for both *E. faecium* and *E. faecalis* isolates (Fig. 3).

**Fig 6:**
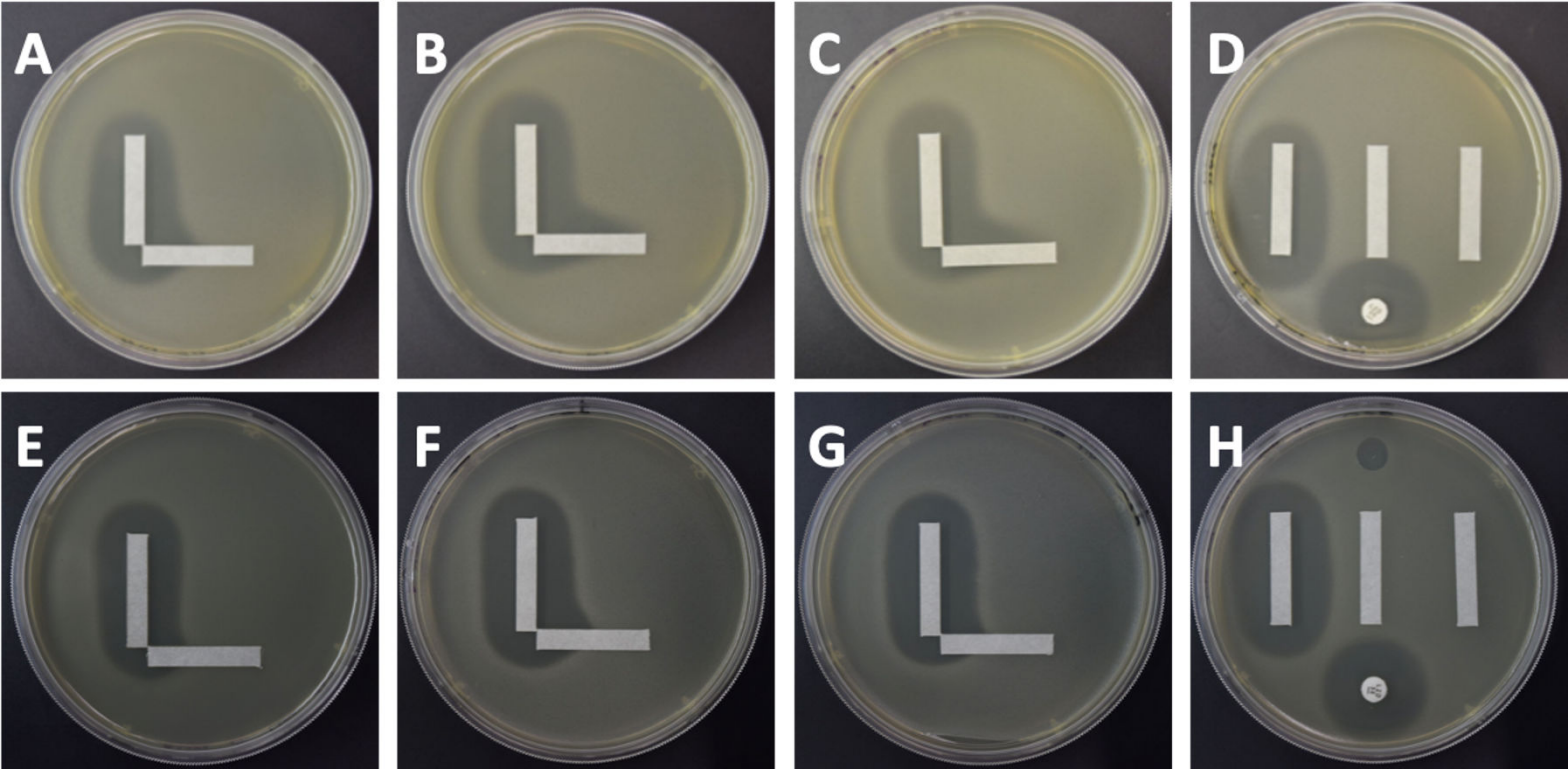
Solid media cooperativity assays for VRE. Each specimen was tested with linezolid (vertical strip) and a phage (horizontal strip). *E. faecium* NYU with phage Bob is demonstrated in panels A–D, where A–C represent three separate replicates of the cooperativity assay, and panel D represents the control plate with a vertical linezolid strip (left), blank strip (middle), phage strip (right), antibiotic disk (bottom), and phage spot (top). *E. faecalis* B3286 with phage PL is demonstrated in panels E–H, where panels E–G represent separate replicates and panel H represents the control plate.

We also evaluated whether a second class of antibiotics against VRE isolates demonstrated cooperativity with phages. We used *E. faecium* NYU in combination with linezolid and phage Bob (myovirus). In each of the replicates, we identified interactions that matched the synergy model (Fig. 6, panels A–D). We identified similar results for *E. faecalis* B3286 with phage PL (siphovirus), indicating that multiple different *Enterococcus* spp. can demonstrate similar results even with different phages (Fig. 3).

**Fig 7:**
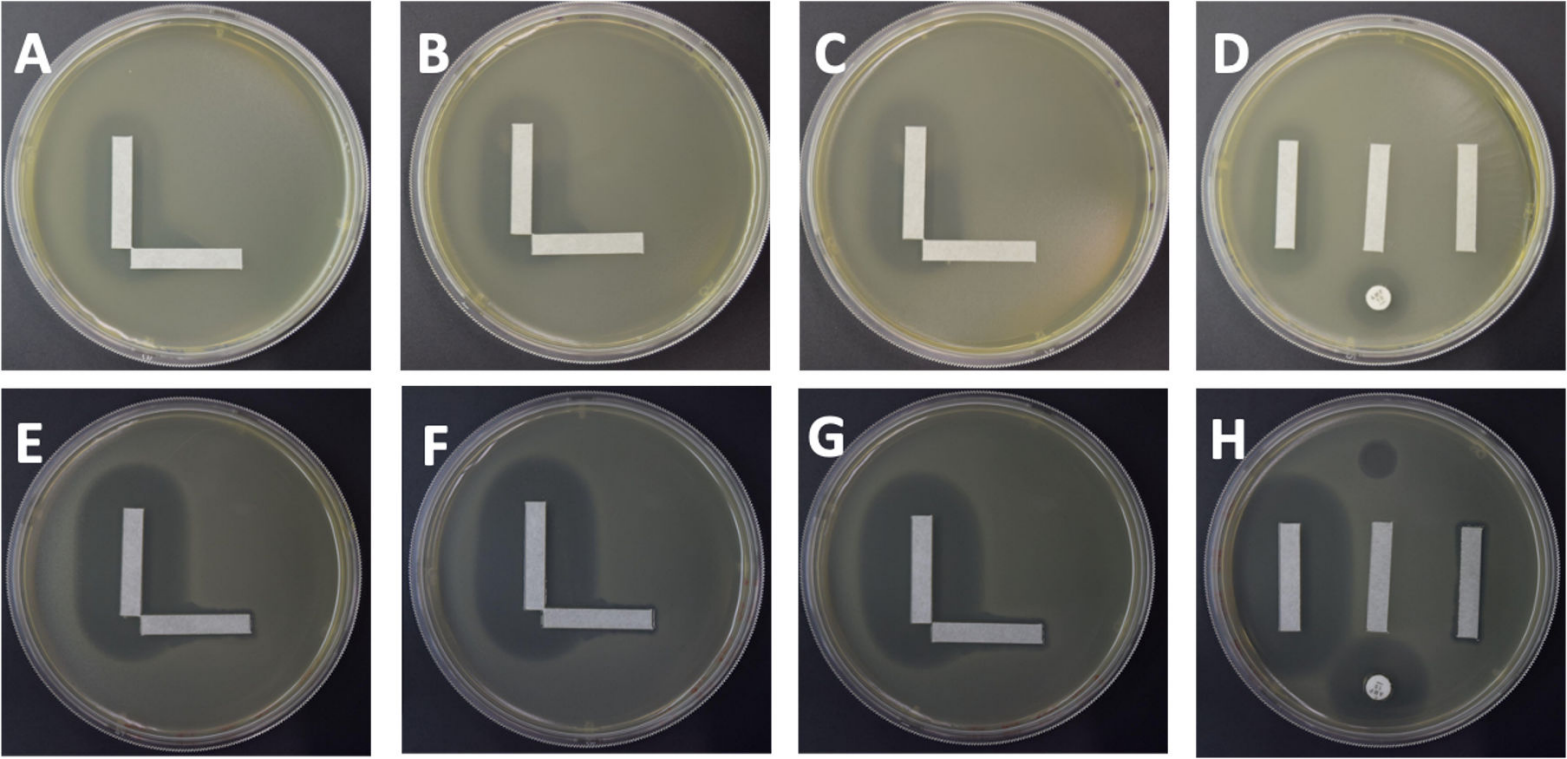
Solid media cooperativity assays for VRE. Each specimen was tested with ampicillin (vertical strip) and a phage (horizontal strip). *E. faecium* NYU with phage Bob is demonstrated in panels A**–**D, where A**–**C represent three separate replicates of the cooperativity assay, and panel D represents the control plate with a vertical ampicillin strip (left), blank strip (middle), phage strip (right), antibiotic disk (bottom), and phage spot (top). *E. faecalis* Yi-6 with phage Bop is demonstrated in panels E**–**H, where panels E–G represent separate replicates and panel H represents the control plate.

We also performed the same cooperativity assay with a beta-lactam antibiotic. Because *E. faecium* is intrinsically resistant to most beta-lactam antibiotics, we performed this assay using ampicillin along with phage Bob (myovirus). We also observed a significant interaction at the intersection of the antibiotic and phage, indicating the presence of synergy (Fig. 7, panels A–D). These data suggest that although *E. faecium* isolates are resistant to certain antibiotics, the combination of these antibiotics with phages can lead to much greater killing. *E. faecalis* often is not resistant to beta-lactam antibiotics such as ampicillin. We also noted significant synergistic interactions when phage Bop (myovirus) was used in combination with ampicillin (panels E**–**H). These data suggest that there may be common mechanisms that lead to antibiotic/phage synergistic interactions for VRE isolates regardless of the antibiotic class used. A more detailed study will be necessary to uncover the basis by which the synergy occurs between these separate antibiotics and phages.

We performed cooperativity assays for a number of different VRE and VSE isolates of *E. faecium* and *E. faecalis*. These assays were performed using antibiotics ampicillin, vancomycin, and linezolid, but also were performed with different myovirus and siphovirus phages infectious for *Enterococcus* spp. We identified a number of isolates in which no evidence of cooperativity could be identified (Fig. S5). For example, no interactions could be identified for *E. faecium* strain EF208PII nor *E. faecalis* EF140PII. However, there were significant interactions identified not only for *E. faecium* isolates, including EF98PII and NYU (Table S9), but also for *E. faecalis* strains V587, EF116PII, Yi-6, and B3286. In all our analyses of the patterns of interactions between antibiotics and phages, we did not observe any that matched the models of additivity nor antagonism.

### Evaluation of cooperativity in gram-negative STM

**Fig 8:**
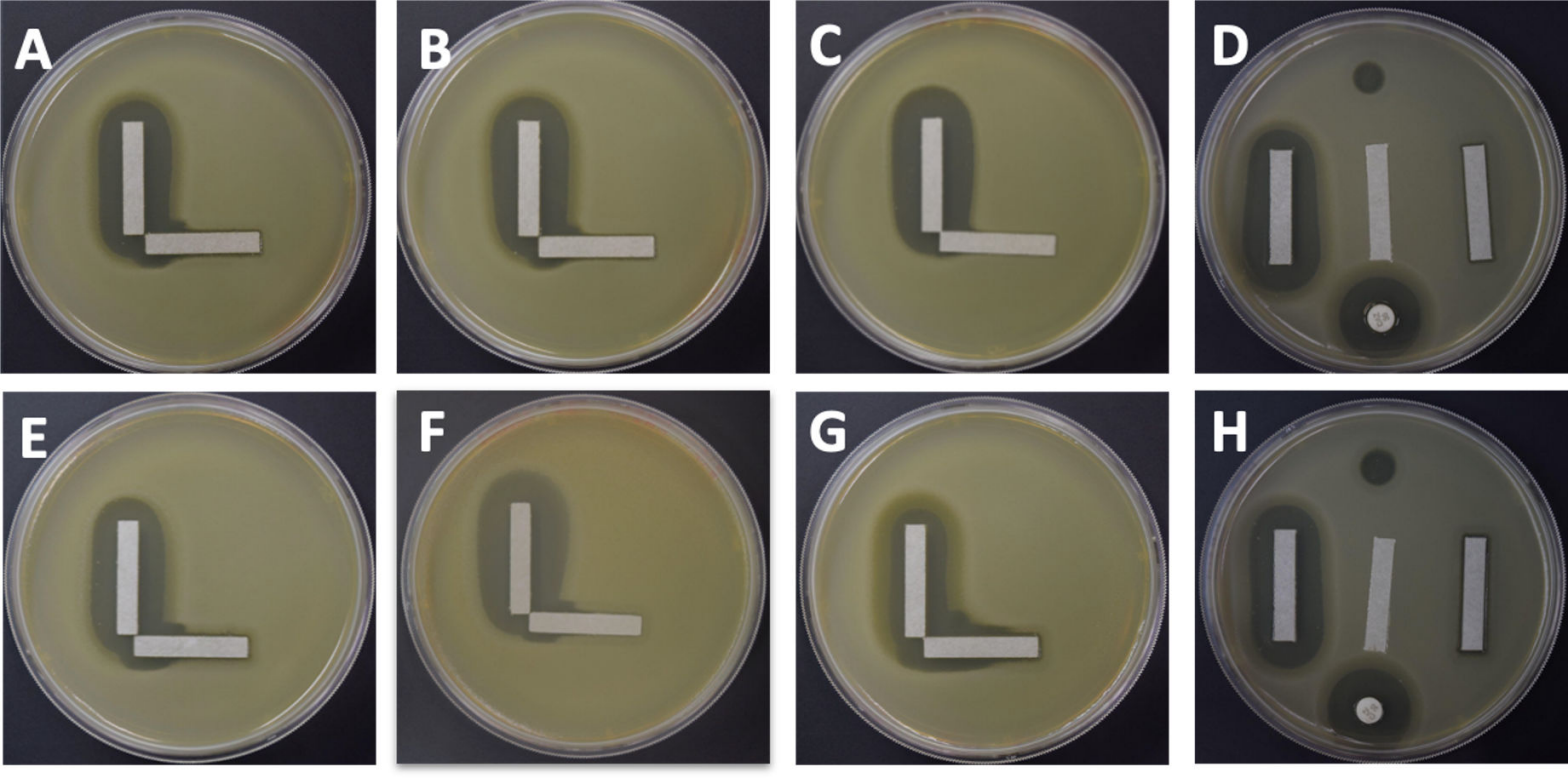
Solid media cooperativity assays for STM. Each specimen was tested with ceftazidime (vertical strip) and a phage (horizontal strip). STM B28B with phage KB824 is demonstrated in panels A–C, which represent three separate replicates of the cooperativity assay. Panel D represents the control plate with a vertical ceftazidime strip (left), blank strip (middle), phage strip (right), antibiotic disk (bottom), and phage KB824 spot (top). STM B28S with phage KB824 is demonstrated in panels E–G, which represent separate replicates. Panel H represents the control plate with a vertical ceftazidime strip (left), blank strip (middle), phage strip (right), antibiotic disk (bottom), and phage KB824 spot (top).

We also analyzed a gram-negative bacterium to identify whether we could observe the same type of synergy that we observed in Enterococcus between antibiotics and phages. We chose the gram-negative bacterium STM because of its profiles of antibiotic resistance, where treatment is often limited to a few antibiotics, including ceftazidime, levofloxacin, and trimethoprim/sulfamethoxazole (Table S6) (25). We first tested ceftazidime along with phage KB824 in our cooperativity assay (Fig. 8, panels A**–**D). We identified substantial evidence of synergistic interactions in all replicates tested. We also noted this type of synergistic interaction extended to additional STM strains B28S (Fig. 8, panels E**–**H) and K279a (Fig. 3; Table S9). We also tested several different phages, which were active against our group of STM isolates (Table S10). The synergy results were not phage specific, as we identified synergistic interactions for a podovirus (KB824) and a siphovirus (ANB28). However, in experiments using the antibiotic levofloxacin, none of the STM isolates demonstrated evidence of cooperativity with phages (Fig. S6; Table S11). In summary, although we identified some instances of synergistic interactions between ceftazidime and different phages, most of our STM isolates did not show any evidence of cooperativity between antibiotic and phage.

## DISCUSSION

Cooperativity between antibiotics and phages can be difficult to measure and has only recently started to garner greater attention (26–28). In its current state, phages are most often administered concurrently with standard-of-care antibiotics to patients with ARB infections under single patient Investigational New Drug Applications. Because of concurrent antibiotic use, it is often difficult to discern whether the antibiotics, the phage, or the combination of both resulted in improvement. There have been anecdotal cases that demonstrate the potential for cooperative interactions between antibiotics and phages (12, 29), and sophisticated laboratory methods for synergy testing in broth (9), but there are no standardized techniques by which cooperativity is measured. Furthermore, synergy for antimicrobials is generally performed in clinical microbiology facilities (30). Liquid media synergy assays are too complicated to be performed routinely in most clinical laboratories. We developed this solid media cooperativity assay because its simplicity may allow for it to be used broadly across clinical microbiology facilities. Although there may be more precise methods we could develop for characterizing cooperative interactions between antibiotics and phages, the simplicity of the assay we have developed could allow for its adoption across laboratories without the need for expensive equipment. Cooperativity could include multiplicative synergy but could also include additive cooperativity. In this paper, we were careful to use the term cooperativity generically, until we could provide evidence that the relationships we were observing actually represented synergy.

The development of a simplistic assay that can be performed in clinical microbiology laboratories across the globe is important for the future of phage therapy and in particular the use of phage/antibiotic combination therapy. Right now, in most cases, it is required that standard-of-care antibiotic therapy is delivered along with phages when phages are given to patients who are treated with phages (29), yet very little is known about whether the phages and antibiotics work together to eliminate the causative pathogens of the illness. Assays such as the one developed here offer the ability to make rational choices about antibiotic and phage combinations because those combinations can be tested *in vitro* in a rather simple manner prior to delivery to the patient. By not requiring the acquisition of expensive equipment, this assay is instantly more assessable for clinical facilities than the more complex broth-based assays. The next step in the development of these assays will be to determine k-values that potentially correlate with treatment successes and use that data to better inform treatment choices in the future.

It was important in the development of this solid media cooperativity assay that we formulate a process that can work for a wide variety of microbes, including gram-positive and gram-negative organisms. There is already a body of literature that suggests such cooperativity, at least in liquid media, may occur (9). In the validation of this assay, we chose to focus on VRE isolates because prior studies have suggested that cooperative interactions can be observed (31). Our data extend those findings to solid medium. The antibiotic-resistant nature of VRE makes it an ideal candidate for our analysis because it can cause deep and long-lasting infections that require alternative therapies such as phages (32). We also evaluated STM as an example of a gram-negative organism, as its antibiotic-resistant nature significantly limits antibiotic treatment options (33). STM also is capable of causing long-lasting infections due to its ability to infect those in the cystic fibrosis population, where the organism can be incredibly difficult to eradicate (34). Our finding of synergistic interactions between phages and the antibiotic ceftazidime may restore the ability to use this antibiotic for these STM infections, where we observed synergy largely in STM isolates that showed intermediate MICs to ceftazidime alone. Future work will be necessary to determine how the phage may restore the susceptibility to ceftazidime, and it may be through reduced expression or efficacy of the L1 and L2 beta-lactamases, or via changes in cell wall composition in response to the phage. We hypothesize that synergistic interactions between antibiotics and phages are not limited to the Enterococcus and STM isolates used in this study but can likely be extended to further ARB such as the ESKAPE pathogens that are often the target of phage therapies.

Identifying synergistic interactions in an *in vitro* study such as this does not necessarily predict what may occur when such treatments are utilized *in vivo*. However, prior studies have indicated that *in vitro* responses may predict the utility of such treatments in humans (35). Even though antibiotics and phages are used together in the majority of phage therapy clinical cases, the combination has been understudied to date (36). We hope to alter this standard approach by implementing an easy-to-perform assay for identifying phage–antibiotic synergy. Thus, as an increasing number of phage therapy cases take place, physicians can be provided with data to better inform their decisions on whether antibiotics and phages may have cooperative effects.

Anecdotal studies indicate that the administration of both vancomycin and phages may have synergistic activity against VRE (31). Although the mechanisms behind such interactions have not been well studied, our data help to confirm those findings and extend them to an easy-to-perform solid media assay. The currently used broth-based assays are cumbersome and require specific equipment, which makes widespread adoption in clinical laboratories difficult.

We show that there are synergistic interactions between vancomycin and phages with myovirus and siphovirus morphologies (37) for both *E. faecalis* and *E. faecium*. Although we are not aware of specific instances where clinical treatments have taken place for VRE isolates using vancomycin and phages, the *in vitro* data shown here suggest that there is the potential for clinical efficacy. One of the simplest clinical rules available for the treatment of VRE has been to avoid the use of vancomycin (38). Our confirmation of the finding that vancomycin in combination with phages may restore the utility of vancomycin in the treatment of VRE could be of significant benefit in the treatment of this life-threatening pathogen. We identified synergistic interactions for other antibiotics, including ampicillin and linezolid (Fig. 6 and 7), which suggests that a broad array of antibiotics may be available for treatment of VRE when phages are involved, even in cases where the VRE isolates are initially resistant to the antibiotics.

There is a lack of standardization of techniques by which to deliver phage therapy and to choose which antibiotic/phage combinations may be the most efficacious (39). We developed the solid media cooperativity assay presented here with the goal to help standardize techniques for decision-making in phage therapy cases and to allow for a much wider adoption of techniques for identifying cooperativity between antibiotics and phages. Our results indicate that this assay is robust and reproducible, can be extended to both gram-positive and gram-negative bacteria, can be applicable across different phage morphologies, applies to multiple antibiotics, and does not necessarily require pre-existing antibiotic nor phage susceptibility in the target bacteria for cooperativity to be observed. We believe solid media assays for the detection of phage/antibiotic cooperativity should serve as standard adjunctive testing to help guide the use of antibiotics and phages in phage therapy cases.

## SUPPLEMENTAL MATERIAL

Figure S1 spectrum.03221-23-s0001.pdf Stamping procedure.

Figure S2 spectrum.03221-23-s0002.pdf Possible patterns observed.

Figure S3 spectrum.03221-23-s0003.pdf Strip placement.

Figure S4 spectrum.03221-23-s0004.pdf Interactions observed.

Figure S5 spectrum.03221-23-s0005.pdf Cooperativity observed for VRE.

Figure S6 spectrum.03221-23-s0006.pdf Cooperativity observed for *Stenotrophomonas*.

Supplemental Tables spectrum.03221-23-s0007.pdf Table S1 to S11.

ASM does not own the copyrights to Supplemental Material that may be linked to, or accessed through, an article. The authors have granted ASM a non-exclusive, world-wide license to publish the Supplemental Material files. Please contact the corresponding author directly for reuse.
