# Supplementary material for: A simple solid media assay for detection of synergy between bacteriophages and antibiotics
Source: Microbiol Spectr. 2024 Mar 25;12(5):e03221-23. doi: 10.1128/spectrum.03221-23 (PMC11064537; doi:10.1128/spectrum.03221-23)
Supplement: Figure S1 — Stamping procedure. [file spectrum.03221-23-s0001.pdf]

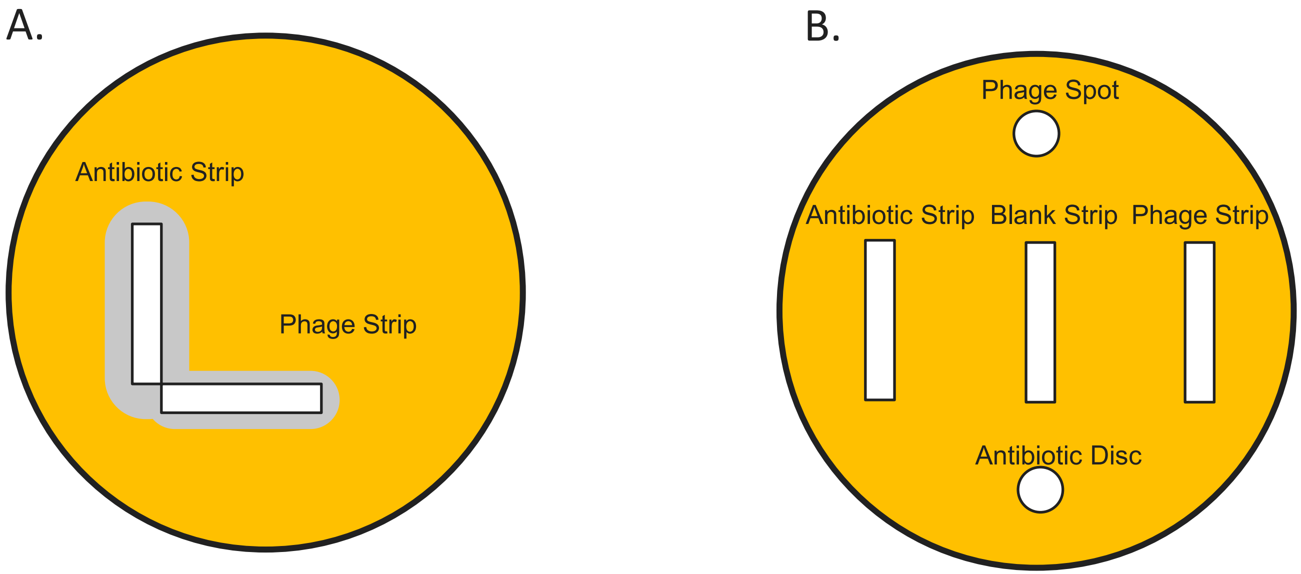

Figure S1. Plate configurations for screening of antibiotic-phage cooperativity. The test plate (Panel A) and control plate (Panel B) configurations are shown.
