# Supplementary material for: A simple solid media assay for detection of synergy between bacteriophages and antibiotics
Source: Microbiol Spectr. 2024 Mar 25;12(5):e03221-23. doi: 10.1128/spectrum.03221-23 (PMC11064537; doi:10.1128/spectrum.03221-23)
Supplement: Figure S2 — Possible patterns observed. [file spectrum.03221-23-s0002.pdf]

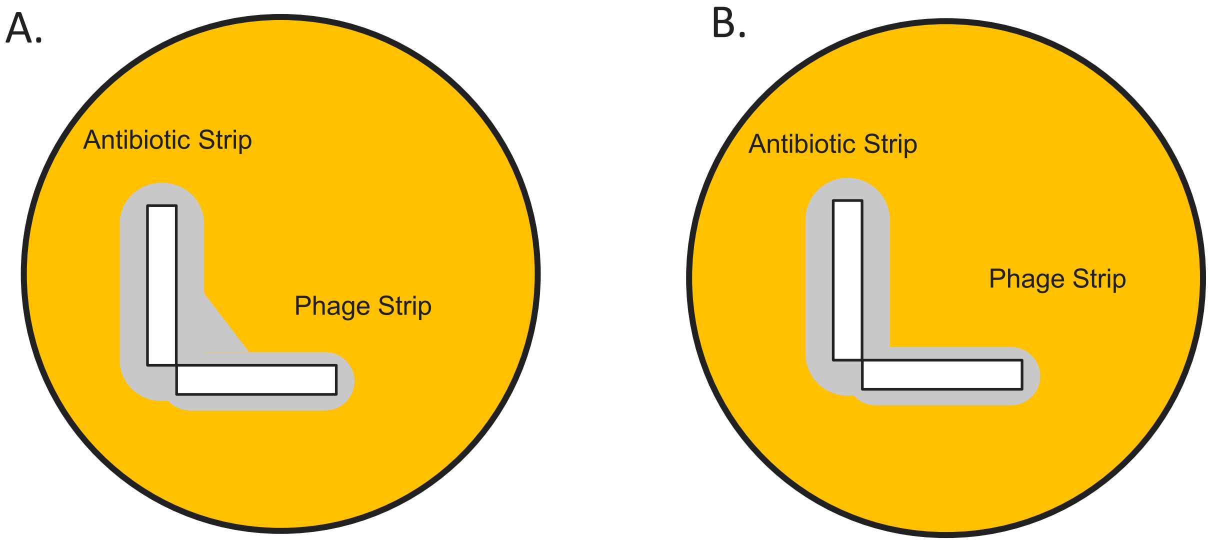

Figure S2. Possible patterns to be observed for phage-antibiotic cooperativity assays. Cooperativity (Panel A), and no cooperativity (Panel B) are shown.
