# Supplementary material for: A simple solid media assay for detection of synergy between bacteriophages and antibiotics
Source: Microbiol Spectr. 2024 Mar 25;12(5):e03221-23. doi: 10.1128/spectrum.03221-23 (PMC11064537; doi:10.1128/spectrum.03221-23)
Supplement: Figure S3 — Strip placement. [file spectrum.03221-23-s0003.pdf]

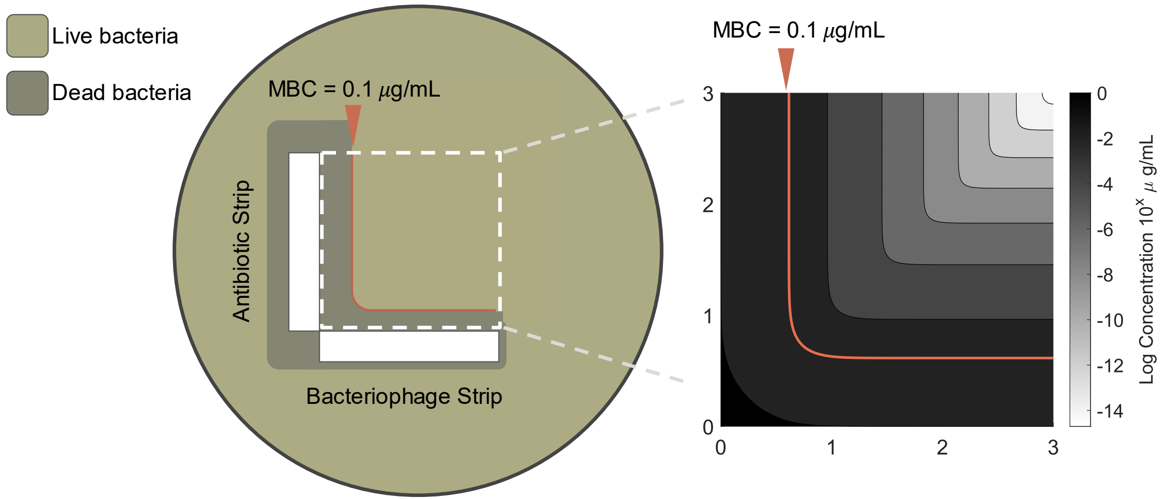

Figure S3. Whatman filter strips loaded with antibiotic or bacteriophage solutions are placed perpendicularly on an agar plate that has an *E. faecium* bacterial lawn. As the antibiotic and phage solutions diffuse through the agar, they interact with bacteria, killing the bacteria in regions where an effective minimum bactericidal concentration (MBC) is reached. The interface between live and dead bacteria creates a profile that aligns with the MBC for the antibiotic, phage, and bacteria combination. A computational model can be used to predict the concentrations of the antibiotic and bacteriophage solutions as they diffuse through the agar. The effective combinatory concentration can be calculated by making assumptions about the antibiotic and phage interactions (*i.e.* no interaction, additive, synergistic, antagonistic).
