# Supplementary material for: A simple solid media assay for detection of synergy between bacteriophages and antibiotics
Source: Microbiol Spectr. 2024 Mar 25;12(5):e03221-23. doi: 10.1128/spectrum.03221-23 (PMC11064537; doi:10.1128/spectrum.03221-23)
Supplement: Figure S4 — Interactions observed. [file spectrum.03221-23-s0004.pdf]

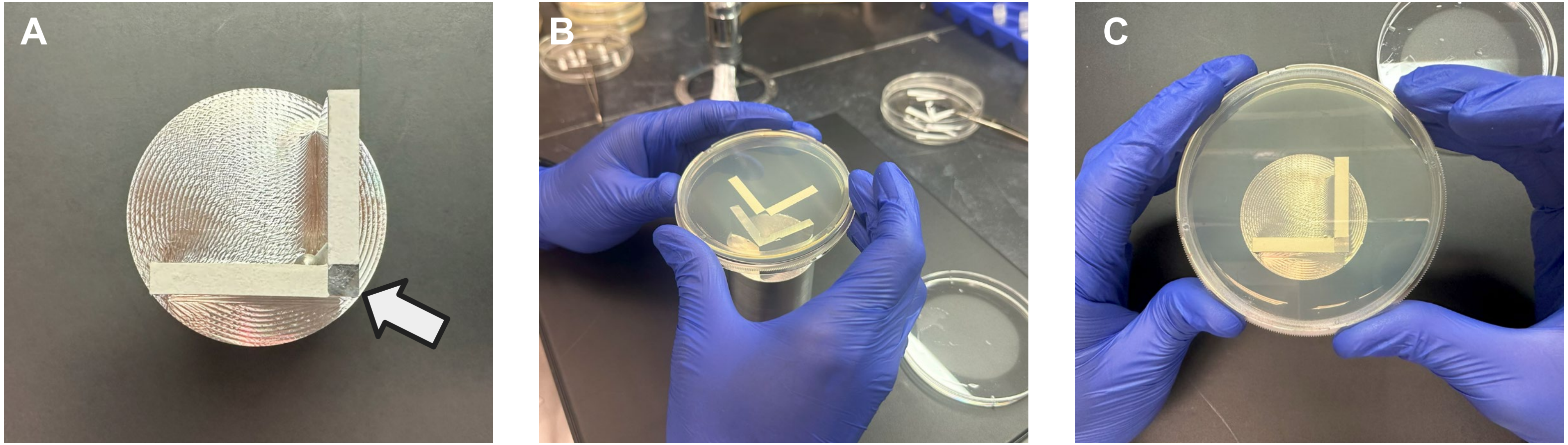


**Figure S4: Stamping Procedure.** A. Dried bacteriophage and antibiotic strips are aligned at 90° using the right-angle edge of the stamp column. The square region marked by arrow indicates that strips are not overlapping and aligned at 90°. B. The solidified plate is inversed and gently stamped onto the aligned strips. C. Top view of the stamping process. L-shape should be stamped so that there is plenty of room for bacteriophage and antibiotic strips to demonstrate proper clearing.
