# Supplementary material for: A simple solid media assay for detection of synergy between bacteriophages and antibiotics
Source: Microbiol Spectr. 2024 Mar 25;12(5):e03221-23. doi: 10.1128/spectrum.03221-23 (PMC11064537; doi:10.1128/spectrum.03221-23)
Supplement: Figure S5 — Cooperativity observed for VRE. [file spectrum.03221-23-s0005.pdf]

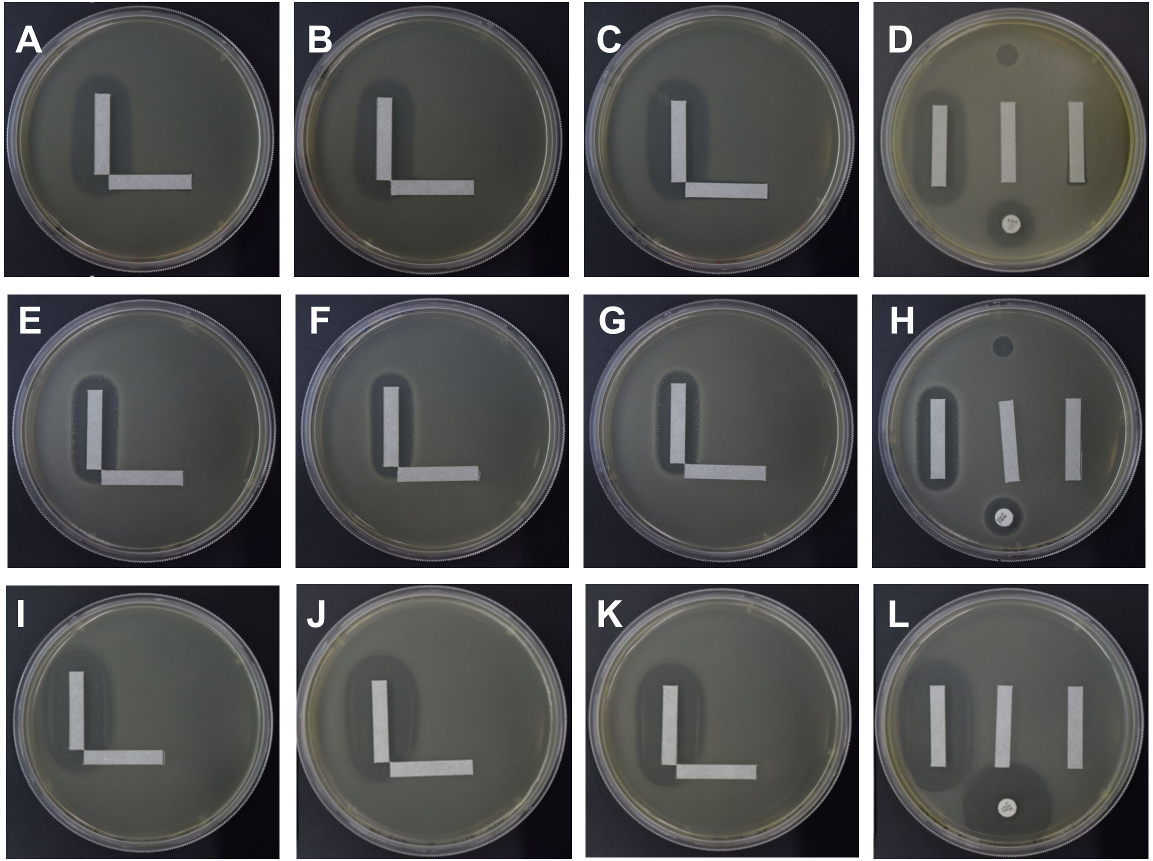

Figure S5. Solid media cooperativity assays for Vancomycin Resistant Enterococcus (VRE). *E. faecium* EF208PII with antibiotic vancomycin and phage PL is represented in Panels A-D. *E. faecium* EF98PII with phage Ben and antibiotic ampicillin is represented in Panels E-H. *E. faecium* EF208PII with phage PL and antibiotic linezolid is represented in Panels I-L. Each specimen was tested with an antibiotic (vertical strip) and a bacteriophage (horizontal strip). Panels D, H, and L represent the control plate with a vertical antibiotic strip (left), blank strip (middle), phage strip (right), antibiotic disk (bottom), and phage spot (top).
