# Supplementary material for: A simple solid media assay for detection of synergy between bacteriophages and antibiotics
Source: Microbiol Spectr. 2024 Mar 25;12(5):e03221-23. doi: 10.1128/spectrum.03221-23 (PMC11064537; doi:10.1128/spectrum.03221-23)
Supplement: Figure S6 — Cooperativity observed for Stenotrophomonas. [file spectrum.03221-23-s0006.pdf]

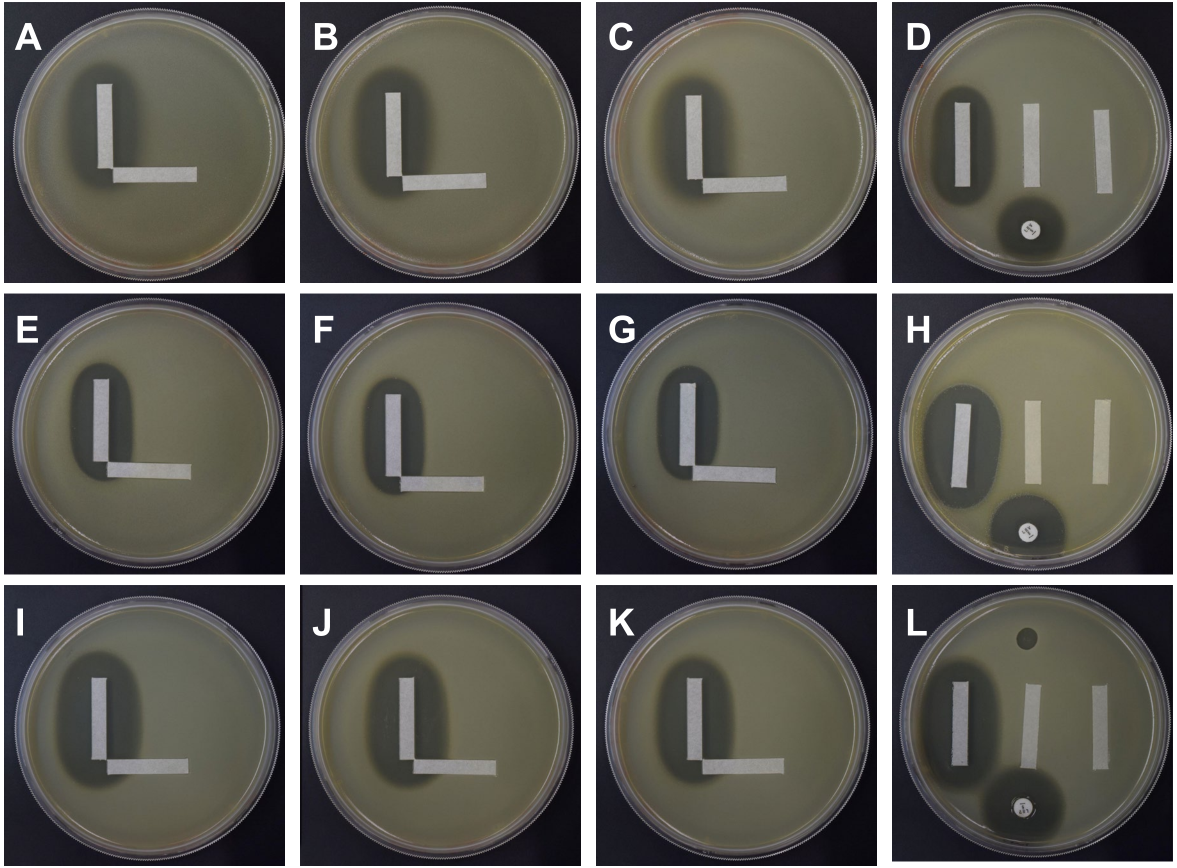

Figure S6: Solid media cooperativity assays for *Stenotrophomonas maltophilia* (STM). Each specimen was tested with levofloxacin (vertical strip) and a bacteriophage (horizontal strip). STM SM17 with phage 2ϕ2 is demonstrated in Panels A-D, where A-C represent 3 separate replicates of the cooperativity assay, and panel D represents the control plate with a vertical levofloxacin strip (left), blank strip (middle), phage strip (right), antibiotic disk (bottom), and phage spot (top). STM SM26 with phage KB824 is demonstrated in Panels E-H, where Panels E-G represent separate replicates and Panel H represents the control plate. STM SM27 with phage ANB28 is represented in Panels I-L.
