# Supplementary material for: A simple solid media assay for detection of synergy between bacteriophages and antibiotics
Source: Microbiol Spectr. 2024 Mar 25;12(5):e03221-23. doi: 10.1128/spectrum.03221-23 (PMC11064537; doi:10.1128/spectrum.03221-23)
Supplement: Supplemental Tables — Table S1 to S11. [file spectrum.03221-23-s0007.pdf]

Table S1: Concentration profiles

Concentration of Individual antimicrobial agents. Antibiotic (α) and Bacteriophage (β)	
$C_\alpha(x,t) = C_0 + (C_{\alpha,source} - C_0) * (1 - \mathrm{erf}\,(\eta_\alpha))$	*Eq. 1A*
$C_\beta(y,t) = C_0 + (C_{\beta,source} - C_0) * (1 - \mathrm{erf}\,(\eta_\beta))$	*Eq. 1B*
Error function	
$\mathrm{erf}(\eta_\alpha) = 2/\sqrt{\pi} \int_0^{\eta_\alpha} e^{-x^2} dx$	*Eq. 2A*
$\mathrm{erf}(\eta_\beta) = 2/\sqrt{\pi} \int_0^{\eta_\beta} e^{-y^2} dy$	*Eq. 2B*
Non-dimensionalized distance	
$\eta_\alpha = \frac{x}{2\sqrt{D_\alpha * t}}$	*Eq. 3A*
$\eta_\beta = \frac{y}{2\sqrt{D_\beta * t}}$	*Eq. 3B*
Boundary Conditions (B.C.)	
$C_\alpha(0,t) = C_{\alpha,source}$	*B.C. 1A*
$C_\alpha(\infty,t) = C_0$	*B.C. 2A*
$C_\beta(0,t) = C_{\beta,source}$	*B.C. 1B*
$C_\beta(\infty,t) = C_0$	*B.C. 2B*
Initial Conditions (I.C.)	
$C_\alpha(x,t \leq 0) = C_0$	*I.C. 1A*
$C_\beta(y,t \leq 0) = C_0$	*I.C. 1B*

Table S2: Modeling parameters

Parameter	Variable	Value	Units
Individual concentration profiles			
$C_\alpha(x,t)$	Antibiotic concentration profile	Eq. 1A	μg/mL
$C_\beta(x,t)$	Bacteriophage concentration profile	Eq. 1B	μg/mL
C_0	Initial concentration	0	μg/mL
$C_{\alpha,source}$	<u>Antibiotic strip concentration</u><br>Vancomycin<br>Linezolid<br>Ampicillin	1.5<br>1.5<br>0.4	μg/mL
$C_{\beta,source}$	<u>Bacteriophage strip concentration</u><br>Phage Ben<br>Phage Bop<br>Phage PL<br>Phage Bob	1.2×10^{-2}<br>3.9×10^{-3}<br>3.8×10^{-2}<br>7.2×10^{-3}	μg/mL
η_α	Non-dimensionalized x-displacement	Eq. 3A	-
η_β	Non-dimensionalized y-displacement	Eq. 3B	-
D_α	Antibiotic diffusivity coefficient in agar	1×10^{-6}	cm^2/s
D_β	Bacteriophage diffusivity coefficient in agar	5×10^{-8}	cm^2/s
x	x-displacement	$0 \leq x \leq 3$	cm
y	y-displacement	$0 \leq y \leq 3$	cm
t	time	$0 \leq y \leq 20$hr	s
Combinatory Antibiotic and Bacteriophage interactions			
C_{NI}	No Interaction effective concentration	Eq. 4	μg/mL
C_{add}	Additive effective concentration	Eq. 5	μg/mL
C_{syn}	Synergistic effective concentration	Eq. 6	μg/mL
C_{ant}	Antagonistic effective concentration	Eq. 7	μg/mL
k	Synergy coefficient	$k_{low} = 1 \times 10^3$	-

Parameter	Variable	Value	Units
		$k_{med} =$ 1 x 10^6<br>$k_{high} =$ 1 x 10^{12}	
q	Antagonistic coefficient	1	-

Table S3: Antibiotic (α) and Bacteriophage (β) interactions

No interaction	$C_{NI} = \max(C_\alpha, C_\beta)$	*Eq. 4*
Additive interaction	$C_{add} = C_\alpha + C_\beta$	*Eq. 5*
Synergistic interaction	$C_{syn} = C_\alpha + C_\beta + k * C_\alpha * C_\beta$	*Eq. 6*
Antagonistic interaction	$C_{ant} = q * abs(C_\alpha - C_\beta)$	*Eq. 7*

Table S4: Bacteriophage strip estimated mass concentrations

Bacteriophage	PFU concentration used in experiment (PFU/mL)	Approximate molecular weight of virus	Estimated mass concentration (μg/mL)
Phage Ben	3.9e8 PFU/mL	194 MDa	1.2×10^{-2} μg/mL
Phage Bop	1.3e8 PFU/mL	194 MDa	3.9×10^{-3} μg/mL
Phage PL	1.27e9 PFU/mL	194 MDa	3.8×10^{-2} μg/mL
Phage Bob	2.4e8 PFU/mL	194 MDa	7.2×10^{-3} μg/mL
Phage KB824	1.0e10 PFU/ml	194 MDa	3.22 μg/mL
Phage 2Φ2	2.28e8 PFU/ml	194 MDa	7.4×10^{-2} μg/mL
Phage ANB28	2.25e9 PFU/ml	194 MDa	7.3×10^{-1} μg/ml

Table S5 – Enterococcus strains and antibiotic susceptibilities

Isolate	Antibiotic	MIC	Interpretation
EF98PII (faecium)	Ampicillin	>8	R
	Daptomycin	4	S
	Gentamicin	<=500	S
	Linezolid	2	S
	Penicillin G	>8	R
	Tetracycline	>8	R
	Vancomycin	>16	R
EF208PII (faecium)	Ampicillin	>8	R
	Daptomycin	4	S
	Gentamicin	<=500	S
	Linezolid	4	I
	Penicillin G	>8	R
	Tetracycline	>8	R
	Vancomycin	>16	R
NYU (faecium)	Ampicillin	>8	R
	Daptomycin	>4	No interpretation
	Gentamicin	<=500	S
	Linezolid	2	S
	Penicillin G	>8	R
	Tetracycline	>8	R
	Vancomycin	>16	R
B3286 (faecalis)	Ampicillin	2	S
	Daptomycin	2	S
	Gentamicin	>500	R
	Linezolid	<=1	S
	Penicillin G	8	S
	Tetracycline	<=5	S
	Vancomycin	1	S
Yi-6 (faecalis)	Ampicillin	0.5	S
	Daptomycin	<=1	S
	Gentamicin	<=500	S
	Linezolid	2	S
	Penicillin G	2	S
	Tetracycline	>8	R
	Vancomycin	1	S
EF116PII (faecalis)	Ampicillin	1	S
	Daptomycin	<=1	S

	Gentamicin	>500	R
	Linezolid	<=1	S
	Penicillin G	4	S
	Tetracycline	>8	R
	Vancomycin	>16	R
EF140PII (faecalis)	Ampicillin	1	S
	Daptomycin	2	S
	Gentamicin	>500	R
	Linezolid	2	S
	Penicillin G	8	S
	Tetracycline	>8	R
	Vancomycin	>16	R
V587 (faecalis)	Ampicillin	1	S
	Daptomycin	2	S
	Gentamicin	>500	R
	Linezolid	<=1	S
	Penicillin G	4	S
	Tetracycline	<=0.5	S
	Vancomycin	>16	R

Table S6 – Stenotrophomonas strains and antibiotic susceptibilities

Isolate	Antibiotic	MIC	Interpretation
**B28S**	Ceftazidime	16	I
	Levofloxacin	>4	R
	Trimethoprim-Sulfamethoxazole	1/19	S
**K279a**	Ceftazidime	16	I
	Levofloxacin	>4	R
	Trimethoprim-Sulfamethoxazole	1/19	S
**B28B**	Ceftazidime	16	I
	Levofloxacin	>4	R
	Trimethoprim-Sulfamethoxazole	1/19	S
**SM12**	Ceftazidime	2	S
	Levofloxacin	2	S
	Trimethoprim-Sulfamethoxazole	<0.5/9.5	S
**SM15**	Ceftazidime	2	S
	Levofloxacin	<=1	S
	Trimethoprim-Sulfamethoxazole	<=0.5/9.5	S
**SM17**	Ceftazidime	16	I
	Levofloxacin	>4	R
	Trimethoprim-Sulfamethoxazole	2/38	S
**SM20**	Ceftazidime	4	S
	Levofloxacin	<=1	S
	Trimethoprim-Sulfamethoxazole	<0.5/9.5	S
**SM22**	Ceftazidime	>16	R
	Levofloxacin	>4	R
	Trimethoprim-Sulfamethoxazole	>2/38	R
	Minocycline	0.50	S
**SM26**	Ceftazidime	1	S
	Levofloxacin	<=1	S
	Trimethoprim-Sulfamethoxazole	<=0.5/9.5	S
	Minocycline	0.38	S
**SM27**	Ceftazidime	>16	R
	Levofloxacin	<=1	S
	Trimethoprim-Sulfamethoxazole	<=0.5/9.5	S

**SM49**	Ceftazidime	8	S
	Levofloxacin	>4	R
	Trimethoprim-Sulfamethoxazole	<=0.5/9.5	S
**SM58**	Ceftazidime	>16	R
	Levofloxacin	4	I
	Trimethoprim-Sulfamethoxazole	<=2/38	S
**SM71**	Ceftazidime	>16	R
	Levofloxacin	<=1	S
	Trimethoprim-Sulfamethoxazole	1/19	S

Antibiotics		Disk Concentration (µg)	Soaking Concentration (mg/ml)
Enterococcus	Ampicillin	10	0.47
	Vancomycin	30	1.40
	Linezolid	30	1.40
Stenotrophomonas	Ceftazidime	30	1.40
	Levofloxacin	5	0.23

Table S7: Antibiotic concentrations of disks and strips used in this study. Disk concentrations represent the manufacturer specifications of the antibiotics loaded onto each disk. Strip concentrations indicate the antibiotic concentrations that were used to soak the filter paper strips. The concentrations used to soak each strip were determined by taking the area of the disks compared to the strips and calculating the appropriate concentration of each antibiotic to reproduce an equal concentration of antibiotics per volume between the disks and the strips.

Enterococcus Strains			Bacteriophage Susceptibility			
			Ben	Bob	Bop	PL
E. faecium	EF98PII	VRE	++		++	
	EF208PII		++		++	
	NYU		++		++	
E. faecalis	V587	VRE	++		+	
	EF116PII			++		++
	EF140PII		++	+		++
	Yi-6	Non-VRE	++	+	++	
	B3286		++	++	++	++

++	+	
Full lysis	Intermediate	No Lysis

Table S8: Bacteriophage susceptibility were measured through visually assessing 4µL bacteriophage spot assays on bacterial lawn. Based on clearing of spots full lysis, intermediate lysis, or no lysis was determined.

Enterococcus Strains			**Ampicillin**				**Vancomycin**				**Linezolid**			
			Ben (M)	Bop (M)	Bob (M)	PL (S)	Ben (M)	Bop (M)	Bob (M)	PL (S)	Ben (M)	Bop (M)	Bob (M)	PL (S)
E. faecium	EF98PII	VRE												
	EF208PII													
	NYU													
E. faecalis	V587	VRE												
	EF116PII													
	EF140PII													
	B3286	Non-VRE												
	Yi-6													

Coo

No

(S) - Siph

Table S9: Cooperativity across *E. faecium and E. faecalis* VRE and non-VRE strains in combination with phages Ben, Bop, Bob, and PL in combination with ampicillin, vancomycin, and linezolid. (M) Myoviridae (S) Siphoviridae.

Stenotrophomonas *Strains*		Bacteriophage Susceptibility		
		KB824	2Φ2	ANB28
Stenotrophomonas maltophilia	B28B	++	++	++
	B28S	++	++	++
	K279a			++
	SM12	++	++	++
	SM15	++		++
	SM17			
	SM20			++
	SM22	++		
	SM26			++
	SM27	++		+
	SM49	++		
	SM58			
	SM71			
		++	+	
		Full lysis	Intermediate	No Lysis

Table S10: Bacteriophage susceptibility were measured through visually assessing 4µL bacteriophage spot assays on bacterial lawn. Based on clearing of spots full lysis, intermediate lysis, or no lysis was determined.

Stenotrophomonas Strains		Ceftazidime			Levofloxacin		
		KB824 (P)	2Φ2 (S)	ANB28 (S)	KB824 (P)	2Φ2 (S)	ANB28 (S)
Stenotrophomonas maltophilia	B28B	Cooperativity	No cooperativity	Cooperativity	No cooperativity	No cooperativity	No cooperativity
	B28S	Cooperativity	No cooperativity	Cooperativity	No cooperativity	No cooperativity	No cooperativity
	K279a	No cooperativity	No cooperativity	Cooperativity	No cooperativity	No cooperativity	No cooperativity
	SM12	No cooperativity	No cooperativity	No cooperativity	No cooperativity	No cooperativity	No cooperativity
	SM15	No cooperativity	No cooperativity	No cooperativity	No cooperativity	No cooperativity	No cooperativity
	SM17	No cooperativity	No cooperativity	No cooperativity	No cooperativity	No cooperativity	No cooperativity
	SM20	No cooperativity	No cooperativity	No cooperativity	No cooperativity	No cooperativity	No cooperativity
	SM22	No cooperativity	No cooperativity	No cooperativity	No cooperativity	No cooperativity	No cooperativity
	SM26	No cooperativity	No cooperativity	Cooperativity	No cooperativity	No cooperativity	No cooperativity
	SM27	No cooperativity	No cooperativity	No cooperativity	No cooperativity	No cooperativity	No cooperativity
	SM49	No cooperativity	No cooperativity	No cooperativity	No cooperativity	No cooperativity	No cooperativity
	SM58	No cooperativity	No cooperativity	No cooperativity	No cooperativity	No cooperativity	No cooperativity
	SM71	No cooperativity	No cooperativity	No cooperativity	No cooperativity	No cooperativity	No cooperativity

Cooperativity

No cooperativity

(P) - Podoviridae

(S) - Siphoviridae

Table S11: Cooperativity across *Stenotrophomonas* isolates in combination with phages KB824, 2Φ2, ANB28 and antibiotics ceftazidime and levofloxacin.
